# Laurence-Moon-Bardet Biedl Syndrome With Cholelithiasis

**DOI:** 10.7759/cureus.47316

**Published:** 2023-10-19

**Authors:** Safa Kaleem, Sooraj Srirangadhamu Gopu, Lyluma Ishfaq, Sabah Afroze, Maahin Parvez, Gopi Sairam Reddy Mulaka, Vishal Venugopal

**Affiliations:** 1 Internal Medicine, Shadan Institute of Medical Sciences, Hyderabad, IND; 2 Internal Medicine, Stanley Medical College, Chennai, IND; 3 Internal Medicine, Directorate of Health Services Kashmir, Srinagar, IND; 4 Internal Medicine, Osmania Medical College, Hyderabad, IND; 5 Internal Medicine, Department of Human Physiology, St. Martinus University Faculty of Medicine, Willemstad, CUW; 6 Internal Medicine, Bhaarath Medical College & Hospital, Chennai, IND

**Keywords:** hypogonadism, eosinophilia, microalbuminuria (ma), moon facies, retinitis pigmentosum (rp), foot polydactyly

## Abstract

Laurence-Moon-Bardet Biedl syndrome (LMBBS) is a rare autosomal recessive genetic disorder that is most frequently found in children born from consanguineous marriages. The most prominent clinical characteristics of this syndrome include rod and cone dystrophy, nystagmus, central obesity, polydactyly, hypogonadism in males, renal anomalies, developmental delay, ataxia, speech difficulties, and poor coordination. In this report, we describe the case of a 31-year-old male who had the classical clinical features of LMBBS like developmental delay, retinitis pigmentosa, nystagmus, obesity, hypogonadism, and central obesity, presenting with abdominal pain associated with vomiting and tenderness in the right lower quadrant. The patient was diagnosed with cholelithiasis. This case report emphasizes the atypical complication of cholelithiasis due to the underlying syndrome and the need for further research in this area.

## Introduction

An uncommon autosomal recessive genetic condition, Laurence-Moon-Bardet Biedl syndrome (LMBBS), primarily affects the offspring of consanguineous marriages. Every patient diagnosed with LMBBS does not necessarily exhibit the typical pattern of symptoms [1]. When four primary features, or three primary features plus two supplemental features, are present, a clinical diagnosis can be made. Cone-rod dystrophy, polydactyly, obesity, learning difficulties, hypogonadism in men, and renal anomalies are categorized as primary features, whereas speech difficulties, brachydactyly, developmental retardation, polyuria/polydipsia, ataxia, poor coordination, diabetes mellitus, left ventricular hypertrophy, hepatic fibrosis, spasticity, and hearing loss are categorized as secondary features. Short stature, crowded teeth, hypermobile or lax joints, and early osteoarthritis have been documented in addition to these characteristics [2]. Patients with this syndrome typically arrive in their first or second decade of life with visual acuity impairment that progresses to blindness at a young age [3]. Retinitis pigmentosa (RP), a rod-cone variant of progressive pigmentary retinal degeneration, has been the most frequently seen retinal degeneration in this syndrome [4].

## Case presentation

A 31-year-old man, who was previously diagnosed with Laurence-Moon-Bardet Biedl syndrome, presented to the hospital with complaints of right hypochondriac discomfort, a high temperature, and three episodes of vomiting. He started experiencing abdominal discomfort after consuming foods rich in fat for the past few weeks. The discomfort progressed to severe pain in the right hypochondriac region radiating to the umbilical region, which was intermittent and progressive. Then he experienced bouts of high fever associated with three episodes of vomiting. He had a medical history of hypertension and type 2 diabetes controlled with medications.

**Figure 1:**
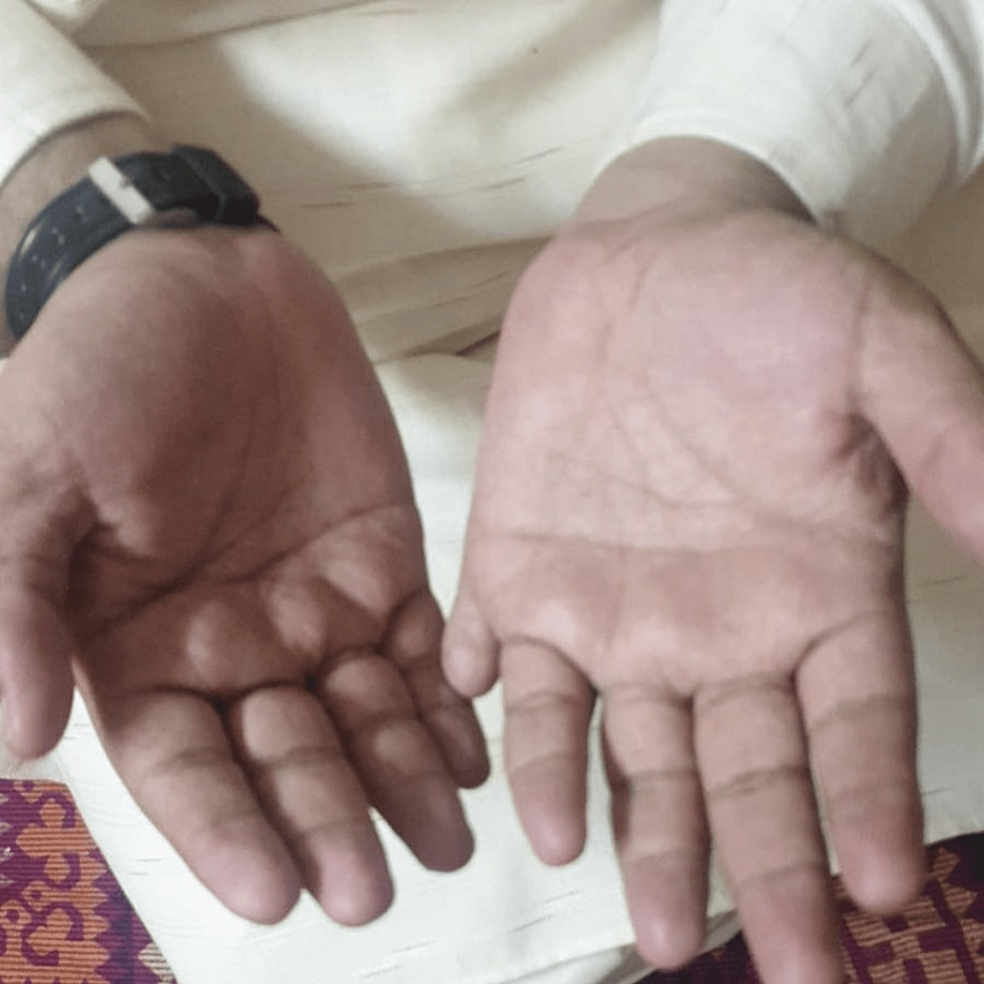
Polydactyly in the upper limb Extra little finger on the right hand

**Figure 2:**
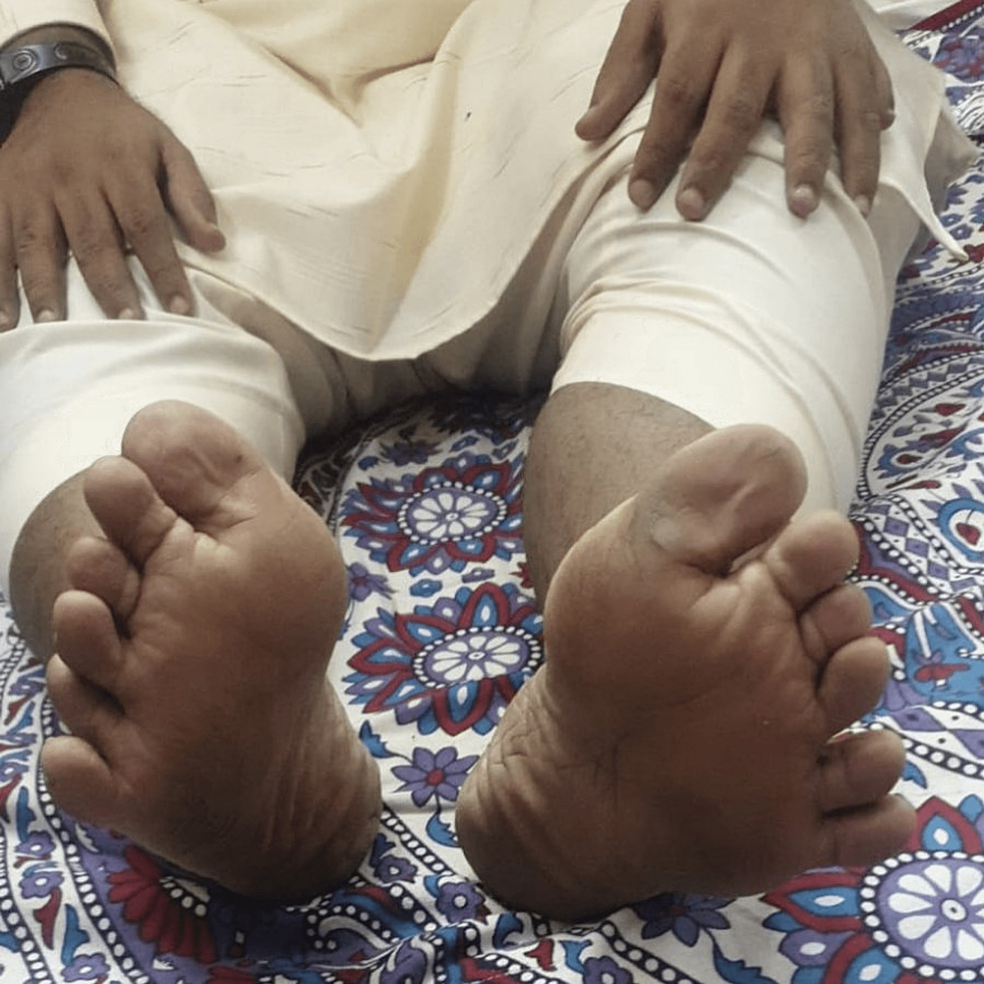
Polydactyly in the lower limbs Extra finger on both feet

His mother stated that he initially had problems with vision in school at the age of seven, for which they consulted an ophthalmologist. There was decreased vision in both eyes and nystagmus on examination, which did not correct even after providing corrective lenses. Fundoscopy showed pigmentary deposits in the peripheral retina associated with mild atrophy, pointing towards retinitis pigmentosa. The patient experienced worsening visual acuity, followed by night blindness, tunnel vision, and eventually vision loss. According to the mother, he had been polydactyly since birth (Figure 1, 2). His mother also stated that he had delayed developmental milestones compared to his siblings. She attested that he was born by normal-term vaginal delivery with a weight of 3.2 kg and out of a non-consanguineous marriage. She stated there were no signs of asphyxia, neonatal distress, or cyanosis after birth. She denied any complications or exposure to any teratogenic substance during pregnancy. The mother denied similar complaints in the family.

Upon examination, the patient was awake, alert, cooperative, and had intact upper and lower limb reflexes. He was obese with a moon-like face. Polydactyly observed in the right upper limb and bilateral lower limbs, frequent blinking of the eyes, unstable gait, and clumsiness. Cardiovascular and respiratory examinations were within normal limits. Abdominal examination revealed a soft, thick anterior abdominal wall with marked tenderness in the right hypochondriac region. There was a lack of pubic and axillary hair and bilateral gynecomastia. Hypogonadism was observed upon genital examination. USG of the abdomen revealed distended gallbladder with evidence of 13.7x5.2mm calculus but normal wall thickness. Lab findings revealed eosinophilia of 14% (normal 1-4), blood glucose of 178 (normal 80-140) and hemoglobin A1c of 7.8 (normal <6). Urinalysis revealed microalbuminuria (normal <30) (Table 1). A dilated fundoscopy was done by an ophthalmologist, which revealed bilateral retinal dystrophy. The MRI of the brain revealed no significant findings.

**Table 1:** Significant lab findings Increased levels of eosinophils and urine albumin, HbA1c and blood glucose. HbA1c: glycated haemoglobin

Test	Findings	Reference range
Eosinophils	14 %	1-4%
Blood glucose	178 mg/dl	80-140 mg/dl
HbA1c	7.8	<6
Urine albumin	53 mg/g	<30 mg/g

Potential risk factors for cholelithiasis in this patient were likely linked to his obesity and glucose intolerance. The patient was started on intravenous fluids, and acetaminophen was administered to control the pain. The patient was advised laparoscopic cholecystectomy. Due to financial constraints, the family denied the procedure. The patient and family were counseled regarding the course, prognosis, and need for surgical management. He was advised to consume a low-fat, low-calorie diet, engage in physical activity, and follow up with a gastroenterologist, endocrinologist, and general physician to manage his condition and prevent any further complications.

## Discussion

LMBBS is a disorder with an identified pentad of symptoms, which are obesity, hypogonadism, intellectual impairment, polydactyly, and retinitis pigmentosa [5]. In order for a child to be affected, both parents must be carriers of the defective gene and must pass it on to the child. There is a one in four probability that a child will be born with LMBBS if both parents carry the defective gene that causes the syndrome. The prevalence rates in North America and Europe range from 1:140,000 to 1:160,000 live births. With an estimated incidence of one in 13,500 and one in 17,500, respectively, in Kuwait and Newfoundland, the rate is significantly higher [6].

Symptoms of LMBBS may start to appear in newborns. The syndrome is typically associated with abnormal antitragus anatomy, syndactyly of the fingers, polydactyly, intellectual impairment, obesity, cryptorchidism, hypoplasia of the penis, renal insufficiency, sensorineural hearing loss, and a short height. Most patients with LMBBS will experience a gradual loss of vision due to retinitis pigmentosa, which begins with night blindness, worsens progressively with loss of color perception, and finally deteriorates into “tunnel vision”. Mild-to-moderate learning challenges attributed to weakened cognitive capacity are common in individuals with LMBBS. However, the suspected disabilities are not due to an underlying visual impairment. A smaller-than-average-size anterior pituitary gland has been observed in people with LMBBS, and as a result, these individuals are susceptible to various complications, including those related to the control of the body’s metabolism, emotional responses to stressors, physical development, low levels of thyroid-stimulating hormone (TSH), decreased levels of estrogen and testosterone, and underdeveloped reproductive organs [7].

No clear diagnostic standards have been established for LMBBS or other PNPLA6-related illnesses due to the vast range of clinical presentations. LMBBS is typically identified during assessments of developmental delays. A definitive diagnosis of LMBBS is made by molecular testing for mutations in the PNPLA6 gene [8].

Patients with LMBBS frequently experience renal issues in addition to the five primary symptoms of this illness. Blood pressure control and frequent testing need to be stressed since there is a high risk of progression to end-stage renal failure, especially in the third and fourth decades of life [9]. In a recent study involving a cohort of 54 Bardet-Biedl syndrome (BBS) individuals, it was shown that urine-concentrating defects may predict the progression of renal insufficiency [10]. Given that our patient exhibited microalbuminuria, it is crucial to conduct regular renal function evaluations and urinalysis as part of the necessary follow-up protocol.

Given the numerous complications associated with this ailment, the patient must have access to a robust, all-encompassing healthcare team. Symptom management is the primary focus of LMBBS. Since there is currently no recognized therapy for LMBBS, early intervention is essential to give children the best chance at a normal life. The management of everyday living skills requires the use of hearing and vision aids, speech therapy, occupational therapy, and hormone therapy. Renal problems are the primary cause of mortality, with life expectancy often being lower than that of the general population. Therefore, early renal screenings and evaluations might be helpful. The coexistence of Laurence-Moon-Biedl-Bardet syndrome (LMBBS) and cholelithiasis is considered rare. While cholelithiasis itself is a relatively common condition, occurring in a significant portion of the population, the coexistence of cholelithiasis with a complex genetic disorder like LMBBS is less common. There are various mechanisms through which obesity is known to increase the chance of developing cholelithiasis. Gallstone development is influenced by the equilibrium between cholesterol and bile salts, which can be disrupted by hypercholesterolemia and eventually excessive cholesterol levels in the bile. Insulin resistance and metabolic syndrome, which are frequently associated with obesity, can impair the liver's ability to handle cholesterol and bile salts. Obesity can also lead to slower gallbladder emptying, causing bile to become concentrated [11].

Several recent studies report an association between hypothyroidism, a frequent finding in LMBBS, and common bile duct (CBD) stones. Lack of thyroxine causes a decrease in liver cholesterol metabolism, which impairs the motility, contractility, and filling of the gallbladder, thereby promoting the retention of cholesterol crystals and the formation of gallstones [12].

The management of cholelithiasis typically depends on the patient’s presenting complaints and the presence of complications and comorbidities. Asymptomatic gallstones may be managed expectantly. With a shorter hospital stay and shorter recovery time than open cholecystectomy, laparoscopic cholecystectomy continues to be the surgical treatment of choice for symptomatic and complicated gallstones. If a patient is unable to have surgery, oral dissolving therapy is only occasionally successful. For individuals with gallbladder empyema and sepsis, percutaneous cholecystostomy is an alternative. Non-steroidal anti-inflammatory drugs (NSAIDs), or narcotic painkillers, are typically used to control pain in the treatment of acute biliary colic [13].

## Conclusions

This is a case with typical features of Laurence-Moon-Biedl-Bardet syndrome, presenting with symptoms suggesting cholelithiasis. Although cholelithiasis is not a common finding in LMBBS, its presence suggests the complex nature of the syndrome. Obesity, which is commonly seen in this syndrome, may contribute to the development of gallstones, emphasizing the need for comprehensive medical management. This case report highlights the importance of a multidisciplinary approach in the management of individuals with LMBBS. It is crucial to remain vigilant in monitoring and addressing potential complications that may arise, even if not immediately associated with the syndrome. This case is an important addition to the growing knowledge base surrounding LMBBS. It underscores the crucial need for ongoing research and clinical awareness to enhance the quality of life for individuals affected by this complex syndrome.
